# Microfluidic-Chip-Integrated Biosensors for Lung Disease Models

**DOI:** 10.3390/bios11110456

**Published:** 2021-11-15

**Authors:** Shuang Ding, Haijun Zhang, Xuemei Wang

**Affiliations:** 1 Department of Oncology, Zhongda Hospital, School of Medicine, Southeast University, Nanjing 210009, China; dingshuang@seu.edu.cn; 2 State Key Laboratory of Bioelectronics, School of Biomedical Engineering, Southeast University, Nanjing 210096, China

**Keywords:** biosensor, microfluidics, organ-on-a-chip, lung model, lung-on-a-chip

## Abstract

Lung diseases (e.g., infection, asthma, cancer, and pulmonary fibrosis) represent serious threats to human health all over the world. Conventional two-dimensional (2D) cell models and animal models cannot mimic the human-specific properties of the lungs. In the past decade, human organ-on-a-chip (OOC) platforms—including lung-on-a-chip (LOC)—have emerged rapidly, with the ability to reproduce the in vivo features of organs or tissues based on their three-dimensional (3D) structures. Furthermore, the integration of biosensors in the chip allows researchers to monitor various parameters related to disease development and drug efficacy. In this review, we illustrate the biosensor-based LOC modeling, further discussing the future challenges as well as perspectives in integrating biosensors in OOC platforms.

## 1. Introduction

### 1.1. Lung Physiology and Diseases

The lungs are among the most important organs in the human body, and are a site for gas exchange. They contain many alveoli with a large total surface area and abundant capillaries wrapped around them. Both alveolar walls and capillary walls are composed of a layer of epithelial cells, which are conducive to gas exchange between the alveoli and the blood. The respiratory membrane is the vital structure for gas exchange, and can be divided into six layers: a liquid layer containing alveolar surfactant, the alveolar epithelial layer, the epithelial basement membrane layer, an interstitial layer between the alveoli and capillaries, the capillary basement membrane layer, and the capillary endothelial cell layer [1]. Common respiratory diseases include inflammation (pneumonia) [2], chronic obstructive pulmonary disease (COPD) [3,4], asthma [5], lung cancer [6,7,8], pulmonary fibrosis, pulmonary embolism, etc. They occur in different anatomical regions (e.g., alveoli or small airways), with varied pathogenesis and therapeutic principles.

### 1.2. Microfluidic Chips

In the 1990s, in order to meet the need for more sensitive, efficient, and rapid separation and analysis of biological samples, Manz and Widmer et al. [9] first proposed the concept of miniaturized total analysis systems (μTASs). Today, such systems have developed into one of the most advanced scientific and technological fields in the world. The core technology is based on microfluidic chip systems, or lab-on-a-chip systems [10]. This refers to the integration of sample preparation, reaction, separation, detection, and other basic operational units involved in conventional chemical and biological fields into a chip of several square centimeters, which has the advantages of rapid detection and analysis, large amounts of information, and high throughput. In recent decades, such systems have been widely used in life sciences, disease diagnosis, and drug screening, becoming among the most popular frontier technologies in the 21st century.

### 1.3. Lung Models

At present, in vitro two-dimensional (2D) cell models/three-dimensional (3D) spheroids, animal models, and human organoid models are commonly used to mimic the lungs, but these models all have their limitations. Monolayer cell culture is simple and cheap, but cannot demonstrate the complex structure and function of the human-organ-specific microenvironment in vivo. 3D spheroids based on hydrogel scaffolds are more similar to the microenvironment, but still have the disadvantages of no perfusion, no stress, and limited vasculature. Animal models, although widely used, are not able to mimic human-specific features, with poor prediction value for patients due to the great differences in respiratory system structure between animal models and humans. Lung organoids can provide a variety of cell types and more complex tissue-specific functions, but they cannot mimic organ-level features of the lungs, such as tissue–tissue interface, epithelial–endothelial crosstalk, and immune cell–host response. These models cannot analyze the recruitment of circulating immune cells under active fluid flow, causing unavoidable problems in the modeling of lung diseases. Therefore, there is an urgent need to develop alternative preclinical models to better mimic the pathophysiology of human lungs. Microfluidic technology is able to handle small volumes of fluids (10^−9^ to 10^−18^ L) across microchannels with dimensions from tens to hundreds of micrometers, which promote the development of human organ-on-a-chip (OOC) systems with the ability to successfully mimic many aspects of the organ-level physiology [11]. A large number of OOC platforms integrated with microfluidic technologies, organ anatomy, physics, materials science, and cell biology have been designed for an array of human organs, including the lungs [12,13,14], liver [15,16,17], gut [18,19], kidneys [20,21], and heart [22,23]. Lung-on-a-chip (LOC) [24], as the first proposed OOC, has always been an appealing research topic for mimicking ALI structure or breathing movement, reducing the awkwardness of lung modeling. LOC is a multifunctional microexperimental platform that can reproduce the key structural, functional and mechanical properties of the human alveolar–capillary interface (ACI, the basic functional unit of living lungs), simulate lung function at the organ level, and reflect the tissue–tissue interface, epithelial–endothelial crosstalk, and immune cell–host response. There have been many reports on the application of lung alveolus-on-a-chip and small-airway-on-a-chip systems in simulating lung inflammation, pulmonary edema, pulmonary fibrosis, viral pneumonia, and lung cancer.

### 1.4. Biosensors

**Figure 1:**
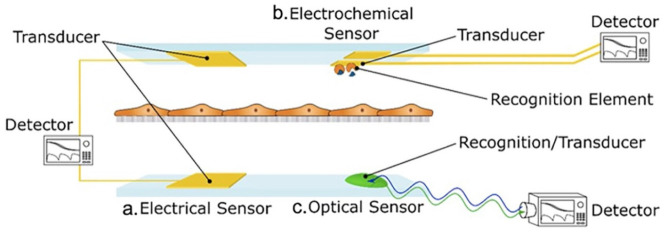
Composition and classification of sensors: Sensors are classified into (a) electrical sensors, (b) electrochemical sensors, and (c) optical sensors. Reproduced with permission from [40].

Biosensors are a kind of chemical sensor. They are a signal analysis tool composed of immobilized biologically sensitive material components (including enzymes, antibodies, antigens, microorganisms, cells, tissues, nucleic acids, etc.), corresponding transducers (including oxygen electrodes, photosensitive tubes, field-effect tubes, piezoelectric crystals, etc.), and a signal detecting device. Biosensors [25] are used to detect biological analytes (e.g., biomolecules), structures, and microorganisms, and can monitor and transmit information about a life process [26,27,28]. As shown in Figure 1, a sensing element (for recognition of biomolecules), signal transducer (for signal translation), and detector (for detecting the signal) make up the sensor. Biosensors were first proposed by Clark et al., who clamped an enzyme solution between two layers of dialysis membrane to form a thin liquid layer, and then glued it to a pH electrode and an oxygen electrode to detect the reaction in the liquid layer. As the lifetime of the enzyme electrode is relatively short, and the purification is also expensive, researchers began to research derivatives of enzyme electrodes—such as animal tissue electrodes, organelle electrodes, and microbial electrodes—which greatly increased the variety of biosensors. According to the sensing principle, the most common integrated sensors can be classified into three groups: electrical [29,30], electrochemical [31,32,33,34,35], and optical [36,37,38]. Electrical signals are often used to deal with cell growth and responses, while optical and electrochemical sensors are commonly used to detect chemical signals. Various integrated biosensors have been used for chemical analysis in microfluidic chips [39]. The basic concept of the reported microfluidic-based biosensor is to integrate the analysis functions required for biochemical analysis on a single chip, including sample preparation, pretreatment, detection, and molecular sorting. The combination of biosensors and microfluidic chips improves analysis capabilities and broadens the range of possible applications. Recently, some efforts have been made to integrate biosensors into OOC platforms as well.

## 2. Biosensor-Free LOC for Lung Modeling

**Figure 2:**
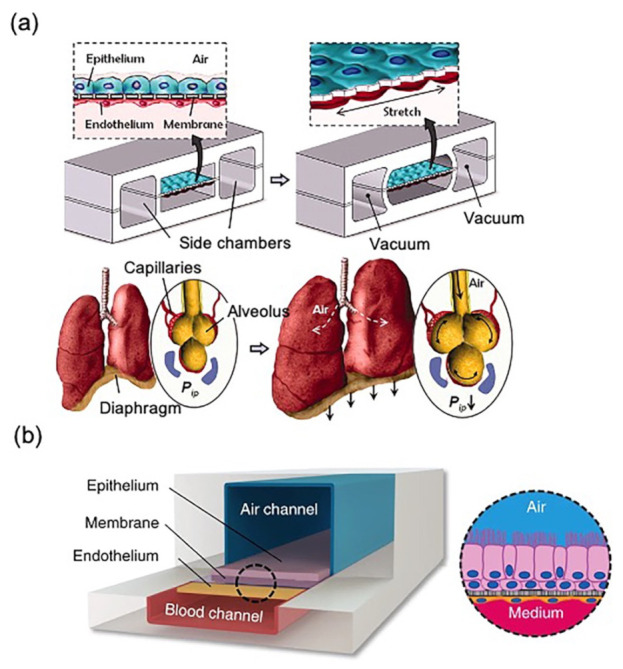
Design of two classical human breathing LOC microdevices: (**a**) Schematic diagram of “alveolar lung-on-a-chip”; physiological breathing movements were reproduced by applying vacuum to the chambers; reproduced with permission from [12]. (**b**) Schematic diagram of the “small airway lung-on-a-chip”; reproduced with permission from [42].

The classical “alveolar lung-on-a-chip” [12] and “small airway lung-on-a-chip” [41,42] were established by Huh et al. and Benam et al., respectively, from the Wyss Institute for Biologically Inspired Engineering at Harvard University (Figure 2). In 2010, Huh et al. [12] produced one of the first biomimetic microfluidic lung models, in which 10 μm of polydimethylsiloxane (PDMS) membrane with ECM was sandwiched between the two PDMS microchannels. ALI was generated for gas exchange with the blood. The upper channel contained epithelial cells while the other channel contained microvascular endothelial cells to mimic the ACI. The membrane between the two channels was forced to deform under a vacuum to pneumatic channels on either side of the membrane. The authors found that ALI culture increased the transbilayer electrical resistance (TER, >800 Ω·cm^2^) and produced tighter ACI as compared to liquid culture conditions. This study became a pioneer for further studies related to LOCs. Benam et al. [41] introduced a human small airway lung-on-a-chip containing mucociliary bronchiolar epithelium and microvascular endothelium; this chip was made of PDMS containing an upper channel and a parallel lower microvascular channel, which were separated by a polyester membrane coated with type I collagen on both sides. Physiological and pathological processes of the lungs were simulated and developed based on this small airway lung-on-a-chip; the authors also modeled several airway diseases [43,44] (e.g., asthma [45], lung inflammation [42], COPD, and COPD exacerbation [13]) on the chip.

In 2012, Huh et al. mimicked pulmonary edema on a chip [46], the structure of which was similar to the alveolar lung-on-a-chip mentioned above. This pulmonary-edema-on-a-chip reproduced lung function in response to interleukin-2 (IL-2), and also successfully screened a drug for pulmonary edema. Zamprogno et al. [47] presented a second-generation LOC with a lung alveolar array based on a biological, thickness/stiffness-controlled membrane made from collagen and elastin via a simple method. Huang et al. [48] used a model of the human alveoli based on physiological structure; it was composed of a 3D porous hydrogel with an inverse opal structure, and then bonded to a PDMS chip. In contrast to traditional PDMS or biological membranes, the inverse opal hydrogel structure is similar to human alveolar sacs, with well-defined, interconnected pores, and can be introduced to LOCs. Zhang et al. [49,50] evaluated the pulmonary toxicity of TiO_2_/ZnO nanoparticles and fine particulate matter (PM2.5) exposure using a novel three-channel 3D LOC model. Hassell et al. [14] created a chip model of human non-small-cell lung cancer (NSCLC) to recapitulate cancer growth, responses to tyrosine kinase inhibitor (TKI) therapy, and dormancy. Xu et al. [51] reported a multiorgan chip with an upstream “lung” and three downstream “organs”, which mimicked the lung cancer metastasis microenvironment. In general, these LOC models mentioned above were biosensor-free, although with different design concepts and applications (see Table 1 for detailed comparisons). Given the biological complexity of the described LOC, future progress must be made in biosensor integration in order to easily monitor related physiological parameters.

## 3. Biosensors in Microfluidic Chips for Lung Modeling

Biosensors have been widely used in the field of analysis, as they can carry out online and continuous monitoring in complex systems, with the properties of high automation, miniaturization, and integration. When biosensors are combined with new approaches, they can have a revolutionary impact on biotechnology. In the following section, we mainly introduce microfluidic chips for lung modeling, along with integrated biosensors for detecting related parameters. Although some microfluidic chips do not have the structure of LOCs, they have successfully achieved the sensing of specific parameters, laying a foundation for the integration of biosensors in LOCs.

### 3.1. Transepithelial Electric Resistance (TEER)

Traditional techniques for the measurement of TEER are not suitable for microfluidic devices that replicate the dynamic microenvironment of lung respiration movements [52]. It is difficult to place the microelectrodes on the chip such that the biomimetic capability of the chip is protected and cells are easily accessed via simple handling.

**Figure 3:**
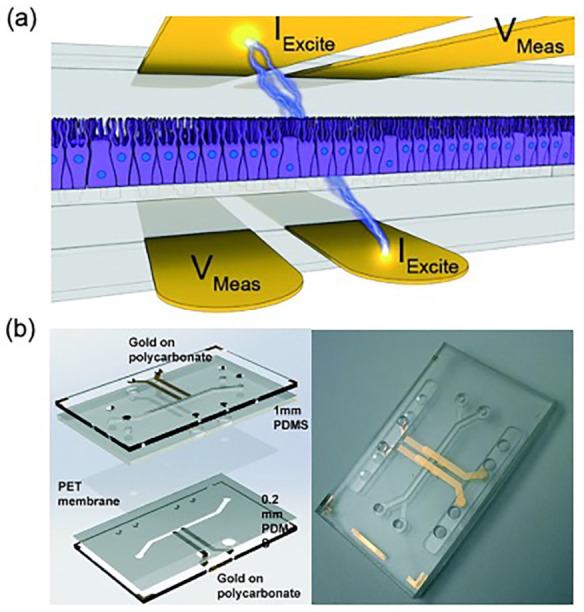
Microengineered human airway-on-a-chip with TEER biosensor: (**a**) Schematic view of the TEER chip’s working principle. (**b**) Photograph of the assembled TEER chip. Reproduced with permission from [53].

In 2017, Henry et al. [53] from the Wyss Institute described a newly developed human airway-on-a-chip with embedded electrodes for TEER biosensors (Figure 3). Four electrodes were integrated into a microfluidic device that they developed previously [42]. The chip was fabricated using polycarbonate (PC) as a base substrate for its high optical clarity, cell culture biocompatibility, ease of machining, compatibility with metal deposition processes, and ease of chemical surface modification. Epoxy moieties were introduced at the PDMS surface using GLYMO prior to the binding of plasma-activated PDMS to the silanol groups introduced at the PC surface via aminopropyltriethoxysilane (APTES) treatment. This method improved bonding efficacy and enabled long-term resistance to hydrolytic cleavage. Electrodes were 1 mm wide, spaced 1 mm apart, and patterned on PC substrates using a laser-patterned, silicon-coated, backing paper shadow mask. These electrodes were not only stable, but also transparent, allowing for real-time monitoring using optical microscopy. The authors successfully maintained the chip for 62 days in culture and 56 days at ALI, without any evidence of cell toxicity from the presence of the gold and titanium layers. This biosensor can be used in OOC modelling of barriers (e.g., the blood–brain barrier), as it enables measurement of barrier function in cultured cells.

**Figure 4:**
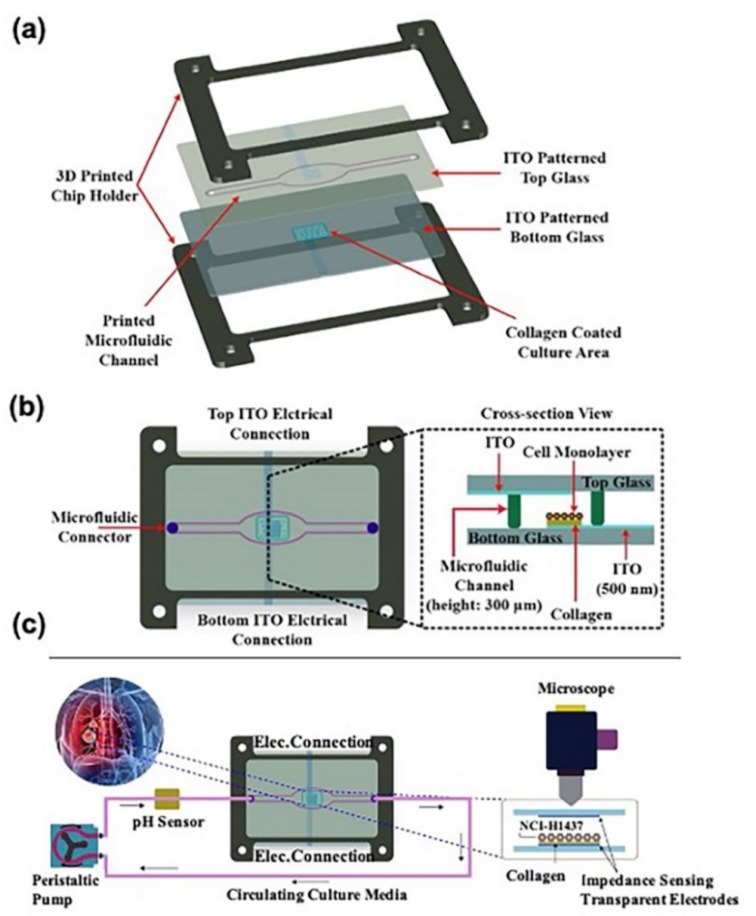
Lung-cancer-on-chip system with multiple sensors: (**a**) Cross-section view and (**b**) top view of microfluidic glass chip fabrication. (**c**) Working flow of the chip for physiological environment monitoring and drug cytotoxicity evaluation. Reproduced with permission from [54].

Increased information output can be obtained by using different types of biosensors in one chip. Khalid et al. [54] introduced a lung-cancer-on-a-chip system equipped with multiple sensors (Figure 4). The chip was prepared in-house by inkjet printing the elastomeric microfluidic channel onto the glass. The top and bottom glasses were held together by a 3D-printed chip holder. During the 54 h real-time monitoring, different concentrations of chemotherapeutics were introduced to NCI-H1437 cells; meanwhile, real-time data of media pH and TEER impedance were obtained via optical pH sensor and top/bottom ITO electrodes. Optical sensors for non-invasive pH monitoring of media were assembled using commercial electronics, white LEDs, optical filters, photodiodes, and 3D printing. The working principle is that when the pH of the extracellular culture medium changes, the color of phenolic red in the culture medium flowing through transparent and biocompatible microfluidic channels changes, and the change in pH can be quantified by measuring the change in light intensity in the channels. Optical pH sensors were characterized and calibrated in the pH range 6.0–8.5 using standard pH media samples. The culture media pH and impedance were monitored for 2 days without any problems in a typical experiment. Then, 500 nm transparent indium tin oxide (ITO)-based TEER impedance-sensing electrodes were patterned using the photolithography technique. The active area of the electrodes was 16 mm^2^. The TEER impedance data converted to the cell index (CI) (normalized impedance values) showed that an increase in the drug concentrations caused higher cell death rates. The authors concluded that increased drug concentration caused medium acidification and higher cell death rates due to an increase in the number of acidic molecules; furthermore, these sensors could also be used in drug screening systems, and the chip introduced by the authors was also a promising tool for the development of personalized medicine.

**Figure 5:**
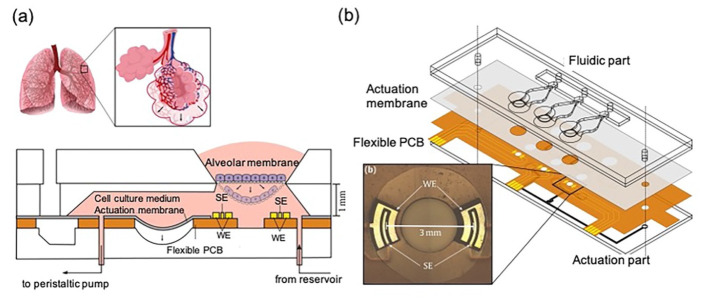
LOC integrated with a MITO system: (**a**) Cross-sectional view of the system. (**b**) Detailed information about the flexible PCB that can be bonded between two layers in the LOC. Reproduced with permission from [55].

Mermoud et al. [55] reported a new micro-impedance tomography (MITO) system with the ability to monitor changes in the lung alveolar barrier at a distance of 1 mm from the electrodes using impedimetric coplanar electrodes (Figure 5). They integrated the system into an LOC that models breathing movement through a thin film, based on their previous study [56]. The sensing system was produced using a printed circuit board (PCB). The electrodes on the PCB consisted of 35 μm of copper covered with an electroless nickel plating and a 50 nm thick layer of immersion gold. The flexible PCB was irreversibly bonded with oxygen plasma between the actuation membrane and the actuation part, providing the LOC with barrier function monitoring in a simple, cost-effective manner.

**Figure 6:**
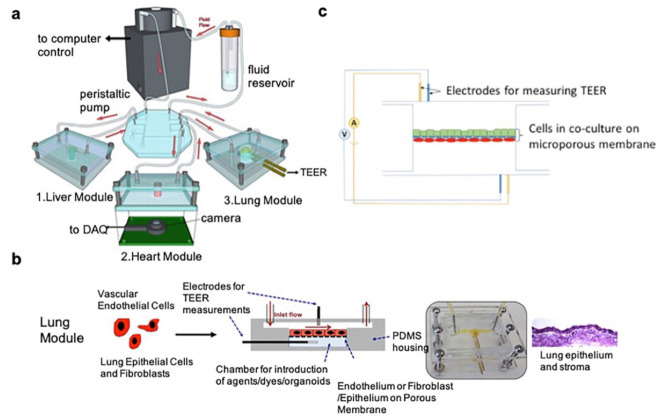
Overall design of the 3-tissue OOC system: (**a**) Illustration of the system. (**b**) Lung modules are formed within microfluidic devices. (**c**) TEER sensors in lung modules are introduced to monitor tissue barrier function over time. Reproduced with permission from [57].

The single-organ LOC can mimic lung cell culture microenvironments, but cannot reproduce the interactions between the lungs and other organs. Combining multiple organ types within a single chip can better model the in vivo microenvironment, and is urgently needed. Skardal et al. [57] described a three-tissue OOC system (liver, heart, and lungs; see Figure 6). Liver and cardiac organoids were integrated into a circulatory perfusion system, which was connected to a lung module with an ALI. Lung modules were composed of endothelial cells, lung fibroblasts, and epithelial cells over a semi-permeable membrane within the chip. Transepithelial resistance (TEER) and short-circuit current (Isc) electrophysiological sensing functions were realized by advanced electrodes. The surfaces of the electrodes were functionalized by immobilizing streptavidin (SPV) on the working electrode via covalent bonding with EDC/NHS. The system was maintained for more than 9 days, with direct monitoring of organoid integrity and organ function. The authors found that bleomycin—a drug that causes lung fibrosis and inflammation—also caused toxicity in the cardiac organoids by releasing inflammatory cytokines, including IL-1β. The advanced in vitro drug screening capability of the system represents an important contribution to the field of drug development.

As the most commonly used type of biosensors in OOCs, electrical sensors measure voltage to determine cell properties and physical properties. Optical sensing relies on various forms of microscopy, without consumption of the analyte. Electrochemical sensors work by catalyzing an analyte into another active product. With the application of sensors in biology, more and more biosensors are used to detect biochemical indicators, and not simply to detect some physical parameters. In the following section, we mainly classify the different sensing targets—such as respiratory viruses, biomarkers (e.g., deoxyribonucleic acid (DNA), ribonucleic acid (RNA), proteins, cells), drug efficacy, oxygen, temperature, etc.—in biosensor-based microfluidic chips for lung modeling.

### 3.2. Respiratory Virus Infections

Since 2019, COVID-19 has been a global pandemic. The current gold standard for diagnosis is viral nucleic acid testing, which is time consuming and labor intensive. Therefore, there is an urgent need for a fast and accurate virus detection method. Qiu et al. [58] reported a dual-functional plasmonic biosensor combining the plasmonic photothermal (PPT) effect and localized surface plasmon resonance (LSPR) to sense transduction for the clinical diagnosis of COVID-19. On the one hand, complementary DNA-receptor-functionalized two-dimensional gold nano-islands (AuNIs) can achieve sensitive detection of the selected sequences from severe acute respiratory syndrome coronavirus 2 (SARS-CoV-2) via nucleic acid hybridization. On the other hand, the AuNIs can enhance the sensing performance by generating thermoplasmonic heat. Jin et al. [59] developed a useful system for the detection of human respiratory adenovirus (HAdV) by combining a biosensor with a microfluidic sample processing module. The detection of viral DNA was accomplished by using a bio-optical sensor of isothermal solid-phase DNA amplification after the DNA was extracted from clinical samples within 30 min using a disposable thin film to facilitate the viral DNA extraction from clinical samples.

### 3.3. Lung Cancer Biomarkers

Biomarkers refer to biomolecules that are signs of normal or abnormal processes, conditions, or diseases found in bodily fluids or tissues. They can be used to monitor the progression and efficacy of a disease—especially in cancer [60,61,62,63,64,65].Biomarkers include DNA, RNA, and proteins (e.g., antigens, cytokines). They are widely detected in bodily fluids such as blood, urine, saliva, tears, and cerebrospinal fluid. Combined detection of multiple tumor markers can improve the sensitivity and specificity of tumor diagnosis. In the following section, biosensors for lung cancer at the molecular level (DNA, RNA, and proteins), organelle level (exosomes), and cell level (circulating tumor cells (CTCs)) are comprehensively discussed.

#### 3.3.1. Molecular Level (DNA, RNA, and Proteins)

DNA [66,67], RNA [68,69], and proteins play important roles in early-stage cancer diagnosis, but their detection remains a challenge because of low expression levels [70,71,72]. Traditionally, cancer-related circulating tumor DNA (ctDNA) is usually detected via personalized analysis of rearranged ends (PARE), whole-genome sequencing, or digital PCR-based methods. MicroRNAs (miRNAs) [73], as a class of small, non-coding endogenous RNAs of ~22 nucleotides long, are traditionally detected by real-time qPCR, Northern blotting, microarray, and deep transcriptome sequencing (RNA-Seq). Lung cancer protein biomarkers [74,75,76]—such as antigens (NSE, SCC, etc.), MMPs, and cytokines—are widely used in clinical practice. Extensive efforts have been devoted to developing ultrasensitive biosensors for the detection of cancer protein biomarkers.

Sheng et al. [77] introduced a dual signal amplification strategy, which was integrated on an electrochemical biosensor for the rapid detection of RNAs (miRNA-17, miRNA-155, miRNA-19b, miRNA-210, thyroid transcription factor-1 messenger RNA (TTF-1 mRNA) and epidermal growth factor receptor (EGFR) mRNA). This platform could selectively and sensitively distinguish early-stage NSCLC patients from healthy controls and benign lung disease patients by identifying low-expression RNA targets in human sera. Portela et al. [78] employed simple colloidal lithography to build a cm^2^-sized nanostructured plasmonic biosensor chip based on nanogap antennas. miRNA-210, a biomarker of lung cancer, was detected by this chip via a DNA/miRNA hybridization assay. The sensing potential was proven to be excellent, owing to a limit of detection (LOD) of 0.78 nM. Aoki et al. [79] fabricated a 384-channel biosensor array chip for the detection of multiple mRNAs and miRNAs for lung cancer. The individual biosensor was composed of a photolithographically fabricated Au/Cr-based electrode modified with peptide nucleic acid (PNA) probes. Sequence-specific responses were proven on the chip with an LOD of 73.3 nM. Furthermore, potential use with polymerase chain reaction (PCR) samples was suggested by PCR-amplified oligonucleotide samples. Zeng et al. [80] developed a novel anchor-like DNA (alDNA) electrochemical biosensor for the detection of Kirsten rat sarcoma viral oncogene (KRAS) point mutation level. Compared to the conventional ligation-based DNA biosensors, the alDNA biosensor was convenient and cheap, with high sensitivity and selectivity; it could capture both wild-type and mutant DNA in one step. Furthermore, mutation detection in blood samples could meet the requirements for early-stage NSCLC diagnosis in clinical settings. Wu et al. [81] developed a chip consisting of gold-coated cover glass and tethered cationic lipoplex nanoparticles (tCLN) containing molecular beacons (MBs), which could capture cancer-cell-derived exosomes or viruses and identify encapsulated RNAs in a single step. The CLNs were able to fuse with the exosomes and form nanoparticle complexes via electrostatic interaction. Then, the MBs hybridized with the target RNAs, and exosomes enriched in the target RNAs were detected by the fluorescence signals of MBs using total internal reflection fluorescence (TIRF) microscopy. Furthermore, only 60 μL of serum and 2.5 h were needed in this system, which showed very promising prospects in the detection of exo-miRNAs and clinical diagnosis.

Chiu et al. [82] constructed a signal amplification sensing film for the detection of the cytokeratin 19 fragment (CYFRA21-1). This novel surface plasmon resonance (SPR) detection assay was ultrasensitive, with an LOD of 0.05 pg/mL in spiked clinical sera, which is 10^4^ times more sensitive than an enzyme-linked immunosorbent assay (ELISA). Cheng et al. [83] developed field-effect transistor (FET) biosensors to detect CYFRA21-1 and neuron-specific enolase (NSE) in both serum and phosphate-buffered saline (PBS); they also integrated two antibody types on the same chip for simultaneous multiplexed detection. Zou et al. [84] introduced a chip cartridge packaged with a Love wave biosensor for the measurement of CEA, NSE, and squamous-cell carcinoma (SCC) antigen in exhaled breath condensate (EBC) collected from both healthy volunteers and lung cancer patients; gold nanoparticles were immobilized onto the biosensor by a sandwich immunoassay. In addition to tumor markers, specific antigens involved in different processes of disease can also help in the diagnosis and prognosis of disease, such as epithelial–mesenchymal transition (EMT) transcription factor, and inflammatory indicators such as C-reactive protein (CRP) and procalcitonin (PCT). Chakravarty et al. [85] demonstrated a silicon chip platform integrated with photonic crystal (PC) microcavity biosensors to detect the EMT transcription factor zinc finger E-box-binding homeobox 1 (ZEB1) in lysates from NCI-H358 cells. The shift in resonance wavelength resulting from the changed refractive index in the PC microcavity could detect the binding of the corresponding antigen. Feng et al. [86] integrated a microelectrode and a cathodic photoelectrochemical (PEC) biosensor into a microfluidic chip for the detection of CYFRA21-1, based on a signal amplification strategy with a detection limit of 0.026 pg/mL.

In addition to the detection of individual biomarkers, multivariate detection has also been widely practiced. Washburn et al. [87] described the simultaneous detection of eight cancer biomarkers (alpha fetoprotein (AFP), activated leukocyte cell adhesion molecule (ALCAM), cancer antigen 15-3 (CA15-3), cancer antigen 19-9 (CA19-9), cancer antigen 125 (CA-125), carcinoembryonic antigen (CEA), osteopontin, and prostate-specific antigen (PSA)) in serum using an antibody-based sandwich assay, in 1 h, based on silicon photonic biosensors. Gao et al. [88] designed a giant magnetoresistance (GMR) multi-biomarker immunoassay biosensor that could simultaneously detect 12 kinds of tumor marker (AFP, CEA, CYFRA21-1, NSE, SCC, PG I, PG II, CA19-9, total PSA, free PSA, free-beta-hCG, and Tg) to screen patients with lung cancer, liver cancer, digestive tract cancer, prostate cancer, etc. The GMR sensor chip was based on a double-antibody sandwich immunoassay method. Gao et al. [89] developed a label-free assay for the multiplexed detection of lung cancer biomarkers (miRNA-126 and CEA) using silicon nanowire field-effect transistor (SiNW-FET) sensors. Integration of the SiNW sensor and PDMS microfluidic device enables rapid, sensitive, and multiplexed detection.

#### 3.3.2. Organelle Level (Exosomes)

Cancer-derived exosomes [90,91,92] with a size of 30-150 nm and density of 1.13–1.19 g/mL have drawn much attention in recent decades, and can be obtained from bodily fluids (such as serum, plasma, or urine). They carry a variety of information on the tumor and tumor microenvironment; thus, they play important roles in tumorigenesis and progression. The detection of such biomarkers is useful for the early diagnosis and drug sensitivity analysis of cancer. Yang et al. [93] presented a novel microfluidic device for the isolation and in situ detection of lung-cancer-specific exosomes collected from patients’ urine. The integrated biosensor was fabricated using poly(methyl methacrylate) (PMMA) and a nonporous gold (Au) nanocluster membrane modified with the capture antibody. The change in scattering intensity due to resonance Rayleigh scattering enables the ultrasensitive detection of exosomes.

#### 3.3.3. Cell Level (Circulating Tumor Cells (CTCs))

CTCs are the tumor cells that are separated from the primary solid tumor and enter the bloodstream for various reasons. They play an important role in early diagnosis, detection of tumor recurrence and metastasis, prognostic evaluation, and treatment guidance. The detection and characterization of CTCs provide a non-invasive approach for monitoring cancer therapy. Chen et al. [94] designed a magnet-deformability hybrid integrated microfluidic chip to enumerate CTCs. NSCLC patient blood samples were used to validate the microfluidic chip clinically, with a high capture efficiency (over 90% at 3 mL/h) and high viability (96%) at high flow rates. Nguyen et al. [95] combined dielectrophoretic (DEP) manipulation and impedance measurement using a single microfluidic device equipped with circular microelectrodes to detect CTCs; the force of DEP and hydrodynamic drag drove A549 lung cancer cells to the center of the working region; the LOD of the impedance biosensor was approximately three cells. The same group [96] also introduced a microdevice with electrical sensors based on aptamer-modified gold electrodes for the detection of A549 cells. This device permitted not only optical microscope observations, but also electrical impedance spectroscopy (EIS) measurements. Nabovati et al. [97] introduced an array of charge-based capacitive measurement biosensors for high-throughput cell growth monitoring; the authors tested both H1299 cells and polystyrene beads, with consistent results with cell-based assays; the results showed that the capacitive electrodes can successfully detect cell attachment and growth. Do et al. [98] combined a DEP microfluidic enrichment platform with a capacitive biosensor to detect CTCs; A549 cells were driven to the working chamber via DEP forces, and then captured by an anti-EGFR modified electrode; finally, cells were detected according to their different capacitance. Li et al. [99] developed a cell isolation microfluidic device based on electrotactic ability; this chip was composed of three parts: a cell immobilization structure, an electric field (EF) generator, and a cell retrieval module. The results show that H1975 cell motility was related to EGFR expression and upregulation of ras homolog family member A (RhoA), regardless of EF stimulation, while it was also related to phosphatase and tensin homolog deleted on chromosome ten (PTEN) expression in the presence of EF stimulation.

### 3.4. Drug Efficacy

Drug efficacy evaluation is important in clinical treatment and drug development. Traditionally, the measurement of cell responses to drugs requires cell counting kit-8 (CCK8), methyl thiazolyl tetrazolium (MTT) assays, animal models, etc., but the biosensor-equipped chip simplifies this process. The corresponding result can be read directly through the biosensor. Pan et al. [100] developed a microgroove impedance sensor (MGIS) for monitoring 3D A549 cell viability in a dynamic and non-invasive manner. Cells were planted in microgrooves for in situ impedance measurement. The proliferation and apoptosis of cells indicated by the change in the living cell number caused an inversely proportional change in the impedance magnitude. The results based on this MGIS platform were very similar to the clinically observed effects of chemotherapy on NSCLC. Noh et al. [101] reported in-air monitoring of in vitro monolayer cells by EIS; two chambers in the chip were separated by a porous membrane, on which EIS electrodes were patterned and A549 cells were cultured. Unlike conventional TEER, electrodes were placed laterally—instead of vertically—to the membrane. This in-air EIS biosensor can enable not only the monitoring of cell population, but also the modulation of tight junctions.

### 3.5. Oxygen and Temperature

Oxygen and temperature are two of the most important physical parameters in the process of cell culture. Although important, they are the most easily ignored parameters, because of the difficulty in simply reading out these indicators in the traditional culture process. Zirath et al. [102] developed two microfluidic devices integrated with oxygen-sensitive, microparticle-based biosensor spots. The microdevice was composed of two glass substrates, onto which sensor microparticles were pipetted directly; an adhesive film containing the fluidic structure bonded the two layers together. Partial oxygen pressures, cellular oxygen consumption rates with varying cell types, flow rates, and cell numbers were monitored.

Temperature changes in cells are closely connected with physiological processes. Temperature measurements are beneficial to the study of cellular mechanisms. Zhao et al. [103] developed a microfluidic chip for cellular temperature monitoring using a platinum (Pt) thermosensor. The chip was positioned in a constant water tank 24 h after cell seeding. The results showed that temperature response to cisplatin differed in different cells. In conclusion, this chip could be applied to study cell physiology and pathology, with the ability to monitor cellular temperature.

In general, microfluidic chips integrated with biosensors for lung disease modeling, with different design concepts and applications, are briefly summarized in Table 2.

## 4. Conclusions and Future Perspectives

Biosensors are a new technology developed by combining biotechnology and electronic technology. They have the advantages of good selectivity, high sensitivity, fast analysis speed, and low cost, and can carry out continuous online monitoring in complex systems. Biosensors also have the advantages of high automation, miniaturization, and integration, which greatly reduce the requirements for the working environment. They are very suitable for field analysis, and have important application value in the fields of biology, medicine, environmental monitoring, food, medicine, and military medicine. The development of biosensors has generally gone through the following three stages: (1) the first generation of biosensors consists of electrochemical electrodes and inactive matrix membranes (dialysis membranes or reaction membranes) with fixed biological components; (2) the second generation of biosensors—biological components directly adsorbed or covalently bound to the surface of the converter—do not need the inactive matrix membrane, and do not need to add other reagents to the sample; (3) in the third generation of biosensors, biological components are directly fixed on the electronic components, and can directly sense and amplify the changes in interface substances, so as to combine biometric recognition and signal conversion processing. Biosensors have been incorporated into OOC platforms for a long time, in order to allow for in situ, real-time, small-volume detection of biochemical parameters with minor disturbances to the system [29,37,104,105,106,107,108,109,110]. In this review, we summarized biosensor-free (Table 1) and biosensor-integrated (Table 2) LOC models, illustrating the chip design and sensing signals of biosensor-integrated LOCs in detail by using examples of related studies. Biosensor research requires interdisciplinary knowledge of microfabrication, microengineering, materials science, chemistry, and biology. Major challenges for the successful integration of biosensors into OOC platforms are their miniaturization [111], biocompatibility, and flexibility. The trends in this field are as follows: (a) integrating more than one biosensor type, allowing for increased information acquisition and an increased feasibility of the model; and (b) increasing the detection ability of precise and personalized clinical testing devices. In summary, microfluidic-based biosensors play an important role in achieving high-throughput, highly sensitive, low-cost analysis. There is still a long way to go in the further development of integrated biosensors in LOCs, until more biosensors are explored and the advantages compared to off-chip assays are fully appreciated. Hopefully, this review will help both biologists and engineers to turn their minds to further development in the integration of biosensors in LOCs. The integration of microfluidic chips and biosensors has overcome the main difficulties in the initial stage of development, such as processing technology and flow control technology. The field is moving into a transformative period, where deeper basic research, extensive application, and in-depth industrialization should be accomplished. It is expected that in the near future, the sensor detection systems in microfluidic chips will replace complex equipment in traditional chemical analysis laboratories, and “personalized laboratories” that can monitor disease-related biochemical indicators will become a reality.

## Figures and Tables

**Table 1:** Literature review of some biosensor-free LOCs. Chip models, structure of ACI, corresponding remarks, and whether respiration movement was observed are listed in the table for comparison.

Chip Models	Structure of ACI	Remarks	RM ^1^	Ref.
Alveolar lung-on-a-chip	Alveolar epithelial cells/PDMS/microvascular endothelium	A pioneer for further studies related to LOC. The authors also introduced pulmonary-edema-on-a-chip to mimic lung function, and screened a new drug for pulmonary edema	Yes	[12]
Small airway lung-on-a-chip	Differentiated mucociliary bronchiolar epithelium/PDMS/microvascular endothelium	Modeled asthma, lung inflammation, and COPD exacerbation on the chip, and also evaluated the therapeutic response on the chip	With ALI structure	[13,41,42]
A chip model of human NSCLC	Similar to alveolar lung-on-a-chip	Recapitulated cancer growth, responses to TKI therapy, and dormancy	Yes	[14]
Second-generation lung alveolar array	Human primary alveolar epithelial cells (hAEpCs)/collagen–elastin membrane/human lung microvascular endothelial cells	Biological, stretchable, biodegradable, and thickness/stiffness-controlled collagen–elastin membrane outperforms PDMS in many ways.	Yes	[47]
Physiologically relevant model of human alveoli	hAEpCs/alveoli-like 3D GelMA hydrogels/human umbilical vein endothelial cells (results with HUVEC only available in the Supplementary Materials)	3D porous hydrogel with an inverse opal structure bonded to a compartmentalized PDMS chip. Investigated the pathological effects of cigarette smoking and SARS-CoV-2 infection	Yes	[48]
Three-channel 3D LOC model	Alveolar epithelial cells/ECM/pulmonary vascular endothelial cells	Evaluated the pulmonary toxicity of TiO_2_/ZnO nanoparticles and PM2.5 exposure	No	[49,50]
Multiorgan lung cancer metastasis-on-a-chip	Human bronchial epithelial and lung cancer cells/PDMS/microvascular endothelial cells, fibroblasts, and macrophages	Upstream “lung” and downstream “brain”, “bone”, and “liver” to mimic the in vivo microenvironment of cancer metastasis	Yes	[51]

^1^ RM refers to respiration movement.

**Table 2:** Literature review of some biosensor-based microfluidic chips for lung disease modeling. Detailed sensing parameters and characteristics of corresponding sensing technology are listed for summary.

Sensing Parameter	Sample	Keywords	Advantages	Ref.
Respiratory virus	SARS-CoV-2	A dual-functional plasmonic biosensor combining the plasmonic photothermal (PPT) effect and localized surface plasmon resonance (LSPR) for sensing transduction	High sensitivity; lower detection limit; cost-effective	[58]
	HAdV	Bio-optical sensor of isothermal solid-phase DNA amplification; a disposable thin film to facilitate the extraction of viral DNA	Low-cost; simplicity; fast (30 min); simple instruments	[59]
DNA/RNA biomarkers	miR-17, miR-155, TTF1mRNA, miR-19b, miR-210	CRISPR/CHDC system; early cancer diagnosis	High sensitivity; low-cost; easy scalability; short assay time	[77]
	miR-210	Large-area nano-plasmonic biosensor; nanogap antennas; customized colloidal lithography process	Simple; low-cost; direct and label-free detection; high sensitivity	[78]
	IGFBP5, EGR3, TFF1 mRNAs, miR-17, miR-21, miR-223	384-Channel, photolithographically fabricated electrode; Au/Cr-based; PNA probes modified	Simple; low cost; simultaneous detection	[79]
	KRAS point mutation	alDNA electrochemical biosensor	High accuracy; convenient, low-cost, and time-saving, with broad dynamic range, and high sensitivity and selectivity	[80]
	miR-21 and TTF-1 mRNA	Tethered cationic lipoplex nanoparticles (tCLN) containing molecular beacons (MBs),	Non-invasive and highly sensitive	[81]
Protein biomarkers	CYFRA21-1	Carboxyl-functionalized molybdenum disulfide (carboxyl-MoS2) nanocomposites; signal amplification sensing film	High specificity	[82]
	CYFRA21-1, NSE	FET biosensor	Simple and rapid; low sample consumption; cheap	[83]
	CEA, NSE and SCC	Tumor markers; clinical EBC samples; gold nanoparticle sandwich immunoassay	Sensitive, specific, and rapid; low cost of time and money; low sample volume	[84]
	ZEB1 in lysates from NCI-H358 cells	Photonic crystal (PC) microcavity biosensors	Duplicate or triplicate analyses; high sensitivity and specificity	[85]
	CYFRA21-1	A microelectrode and a cathodic photoelectrochemical (PEC) biosensor based on a signal amplification strategy	Rapid detection; high selectivity; cost-effectiveness	[86]
	AFP, ALCAM, CA15-3, CA19-9, CA-125, CEA, Osteopontin, PSA	Eight cancer biomarkers in serum; antibody-based sandwich assay	Rapid (1 h) and fully automated	[87]
	AFP, CEA, CYFRA21-1, NSE, SCC, PG I, PG II, CA19-9, total PSA, free PSA, free-beta-hCG, Tg	A giant magnetoresistance (GMR) multi-biomarker immunoassay biosensor; simultaneously detects 12 kinds of tumor markers	High throughput; excellent sensitivity, accuracy, precision, and stability; convenient	[88]
	miRNA-126 and CEA	Silicon nanowire field-effect transistor (SiNW-FET)	Multiplexed real-time monitoring; high sensitivity and selectivity; label-free; low-cost	[89]
Exosomes	Lung-cancer-specific exosomes	Isolation and in situ detection; collected from patients’ urine; nanoporous gold (Au) nanocluster membrane modified with the capture antibody	Fast and ultrasensitive; simultaneous isolation and detection	[93]
CTCs/rare cells	CTCs from NSCLC patient blood	A magnet-deformability hybrid integrated microfluidic chip, validated clinically with a high capture efficiency	Versatile and high-efficiency; size/deformability hybrid	[94]
	A549	DEP manipulation; impedance measurement; circular microelectrodes	Simple; rapid; label-free; low-cost	[95]
	A549	Amine-terminated aptamer-modified gold electrodes; early-stage lung cancer	Simple; cheap; biocompatible	[96]
	H1299 cells	An array of charge-based capacitive measurement biosensors for high-throughput cell growth monitoring	Label-free and real-time detection; high throughput; high sensitivity	[97]
	A549	Guided and captured; electrode immobilized by anti-EGFR	High sensitivity	[98]
	H1975 cell	Composed of cell immobilization structure, electric field (EF) generator, and cell retrieval module	Easy cell manipulation and precise field control	[99]
Drug efficacy	A549	MGIS; dynamic and noninvasive monitoring; 3D cell viability	Real-time; noninvasive; high throughput	[100]
	A549	EIS; in-air monitoring	In situ and real-time monitoring of “air-exposed” cells	[101]
Oxygen	A549, HUVEC, ASC, NHDF	Oxygen-sensitive microparticle-based biosensor spot arrays	Non-invasive, real-time, label-free in situ monitoring of oxygen demands and metabolic activity	[102]
Temperature	H1975	Pt thermosensor; cellular temperature monitoring	Non-disposable and label-free	[103]

## Data Availability

Not applicable.
